# A systematic review of the impact of 7-keto-DHEA on body weight

**DOI:** 10.1007/s00404-022-06884-8

**Published:** 2022-12-25

**Authors:** Nishanthini Jeyaprakash, Sara Maeder, Heidrun Janka, Petra Stute

**Affiliations:** 1 Department of Obstetrics and Gynecology, University of Bern, Bern, Switzerland; 2 Medical Library, University Library Bern, University of Bern, Bern, Switzerland; 3 Department of Gynecologic Endocrinology and Reproductive Medicine, University Clinic of Obstetrics and Gynecology, Inselspital Bern, Friedbühlstrasse 19, 3010 Bern, Switzerland

**Keywords:** 7-Keto-DHEA, 3-Acetyl-7-oxo-dehydroepiandrosterone, Obesity, Weight loss

## Abstract

7-Keto-DHEA has been commercially advertised as a dietary supplement to support weight loss. The objective of the present systematic review it to summarize the evidence supporting the use of 7-keto-DHEA in overweight and obese population. The systematic search was conducted in Medline, Embase, Cochrane Library, CINAHL, Web of Science, Scopus, ICTRP, and ClinicalTrials.gov. Additionally, reference lists of eligible studies were considered, and authors of relevant studies were contacted. Two authors independently screened the studies against the inclusion criteria and assessed their risk of bias. In total, 4 out of 686 studies were included in the review. They all held a low risk of bias. Half of them showed a significant reduction in body weight. One study found a decrease in body fat percentage and another one reported a decrease in BMI. Two studies stated an increase in resting metabolic rate. No serious adverse effects were reported. Various possible mechanisms in favor of weight loss are discussed; however, with the evidence currently available, no clear answer can be given regarding 7-keto-DHEA and weight loss. Further studies need to be conducted to clarify the efficacy and safety of this drug before it can be recommended for therapeutic use.

**Supplementary Information:**

The online version contains supplementary material available at 10.1007/s00404-022-06884-8.

## What does this study add to the clinical work

**Table:** 

7-keto-DHEA has been commercially advertised as a dietary supplement to support weight loss, even though the conducted systematic review could not give a clear answer regarding its efficiency.	

## Introduction

In the last past decades, obesity has turned into a major global epidemic that continues to worsen and challenge the health-care system throughout the world. Economically, the cost of treating obesity and its complications account for a big proportion of health-care spending [1]. Overweight and obesity are defined as excessive fat accumulation, mainly caused by an imbalance between energy intake and energy expenditure [2]. Consequently, to treat obesity, a negative energy balance must be achieved such that stored fat is utilized as an energy source [3]. Many have been trying to lose weight [4] by reducing their energy intake, however, without much success [5]. Recent weight loss programs have been promoting dietary supplements which pharmacologically increase energy expenditure [6]. Nutritional supplements for weight loss are widely available at health food stores, fitness centers as well as over the Internet and their sale has been steadily increasing even though there is little to no scientific evidence regarding their safety and effectiveness in weight loss [4].

**Fig. 1:**
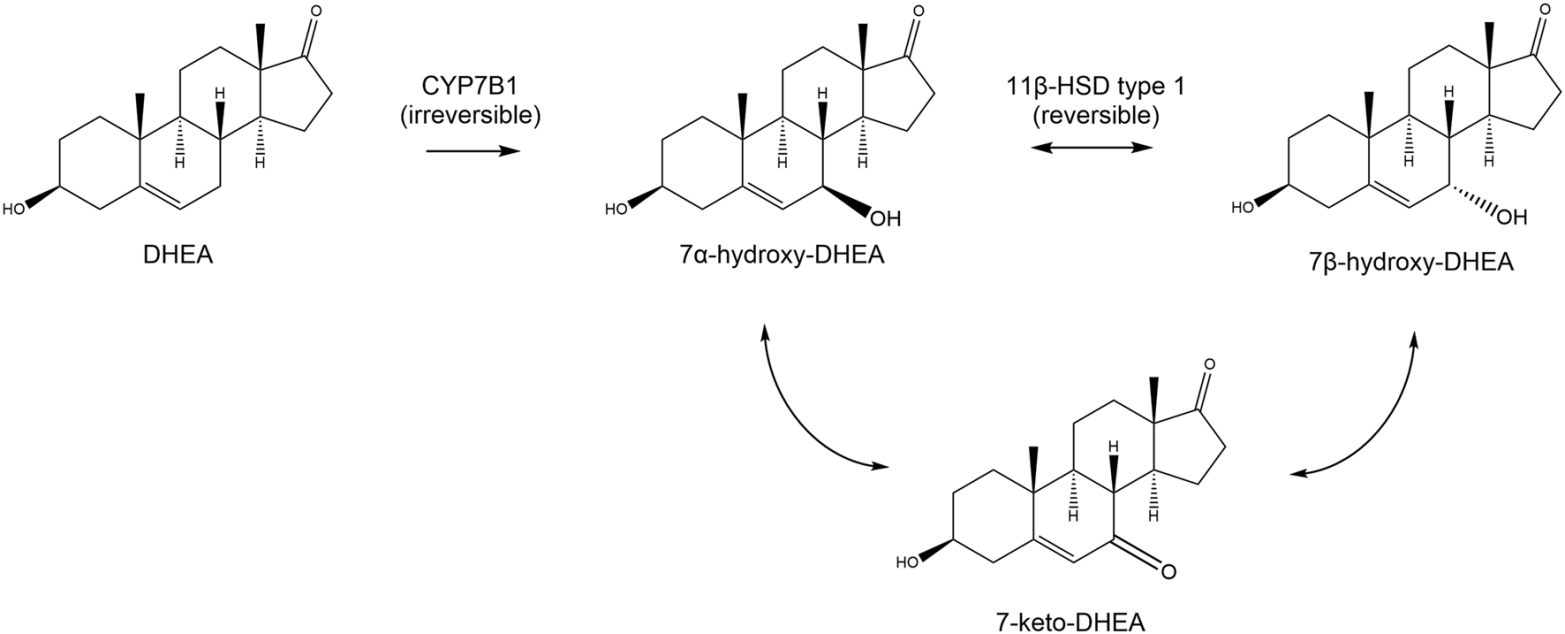
Biosynthesis of 7-keto-DHEA

7-Ketodehydroepiandrosterone (7-keto-DHEA), chemically known under 3-acetyl-7-oxo-dehydroepiandrosterone and shown in Fig. 1, is a naturally occurring metabolite of the steroid hormone dehydroepiandrosterone (DHEA) [7] and is marketed as such an anti-obesity dietary supplement [4]. As shown in Fig. 1, the synthesis of 7-keto-DHEA begins with an irreversible hydroxylation of DHEA in the position C7 by cytochrome P450 7B1 (CYP7B1) under the formation of 7 $\alpha$-hydroxy-dehydroepiandrosterone (7 $\alpha$-OH-DHEA). 11 $\beta$-hydroxysteroid dehydrogenase type 1 (11 $\beta$-HSD 1) then catalyzes the interconversion of 7 $\alpha$-OH-DHEA and 7 $\beta$-hydroxy-dehydroepiandrosterone (7 $\beta$-OH-DHEA) through a 7-keto-DHEA intermediary [8].

An increase in thermogenesis was proposed as a possible mechanism to explain the weight loss effect 7-keto-DHEA is associated with. The elevation in energy expenditure manifests itself through an increase in metabolic rate, consequently leading to fat and weight loss [9]. Many studies showed that DHEA and some of its derivatives are thermogenic and that their ability to induce thermogenesis increases in the following sequence: DHEA–7-$\alpha$-OH-DHEA–7-keto-DHEA–7 $\beta$-OH-DHEA [10]. Their thermogenic properties are likely attributed to increased levels of thermogenic enzymes that shuttle substrate and electrons in and out of the mitochondria and, due to increased proton leak, across the mitochondrial inner membrane. Like thyroid hormone, 7-keto-DHEA increases the levels of mitochondrial sn-glycerol-3-phosphate dehydrogenase (mGPDH) and cytosolic malic enzyme, indicating that they enhance thermogenesis through similar mechanisms [11]. They use a metabolically inefficient pathway called glycerophosphate shuttle for electron transport that bypasses the NADH–ubiquinone sequence of the respiratory chain. The electron transport chain and the mitochondrial oxidative phosphorylation coupled to it are disrupted [12, 13]. The energy released during the electron transfer is not sufficient to uphold the proton motive force across the inner mitochondrial membrane, lowering its membrane potential and dissipating the energy from the proton motive force as heat rather that producing ATP from ADP. To generate the previous proton motive force and the quantity of ATP, the amount of electron transfer must be increased and in consequence more substrate, more oxygen and more stored energy from the cells are consumed [14]. This supports the theory that 7-keto-DHEA promotes fat burning to gain energy by stimulating the fatty acyl CoA oxidase, which is the major enzyme in the oxidation of fatty acids [11].

Another mechanism could be mediated through 11 $\beta$-HSD 1, which catalyzes the conversion of cortisone to active cortisol [8]. Elevated blood levels of glucocorticoids are associated with insulin resistance and obesity [15]. 7-Keto-DHEA is thought to regulate the local cortisol activity through competitive inhibition of this key enzyme, as this favors the production of 7 $\beta$-OH-DHEA over that of active glucocorticoids [8]. In addition, glucocorticoids reduce the number of uncoupling proteins, which could be cancelled out through the possible anti-glucocorticoid property of 7-keto-DHEA. Uncoupling proteins allow protons to enter the mitochondrial matrix without synthesizing ATP; therefore, free energy from the mitochondrial oxidation is converted to heat and again contributes to thermogenesis [9]. 7-Keto-DHEA might also directly elevate the levels of uncoupling proteins [11].

For all these reasons, 7-keto-DHEA has been described as a thermogenic agent with weight loss potential. Bobyleva et al. [11] had reported weight loss in rats after intake of 7-keto-DHEA. The aim of this paper is to conduct a systematic review evaluating the role of 7-keto-DHEA on metabolism and body weight in humans. To our knowledge, no such systematic review has been carried out to date.

## Methods

This systematic review adhered to the Preferred Reporting Items for Systematic Reviews and Meta-Analyses (PRISMA) checklist [16] for the reporting of systematic reviews and is presented in Online Resource 1. The review was not registered, and a review protocol was not prepared.

### Information sources and search strategy

To identify all potentially relevant documents on the topic, literature searches were designed and executed for the following information sources:

- Medline (Ovid) (incl. Epub Ahead of Print, In-Process & Other Non-Indexed Citations, Medline Daily and Ovid Medline Versions) (1946–October 27, 2020)
- Embase (Ovid) (1974–October 27, 2020)
- Cochrane Library (Wiley) (1996–October 27, 2020)
- CINAHL (Ebsco) (1937–October 27, 2020)
- Web of Science (Clarivate) (1900–October 27, 2020)
- Scopus (Elsevier) (1788–October 27, 2020)
- ICTRP Trials Register (WHO)
- ClinicalTrials.gov (NLM)

An initial search strategy in Medline was drafted by a medical information specialist and tested against a list of core references to see if they were all included in the search results. After refinement and consultation, complex search strategies were set up for each information source based on database-specific controlled vocabulary (thesaurus terms/subject headings) and text words. No limits have been applied in any database considering study types, languages, publication years or any other formal criteria. Besides the standard medical bibliographic databases Medline, Embase, Cochrane Library and CINAHL, two international trials registers (ICTRP Trials Register, ClinicalTrials.com) and interdisciplinary search portals (Web of Science and Scopus) were searched. All searches were run on October 28th, 2020. In addition to electronic database searching, reference lists and bibliographies from relevant publications were checked to identify any further reports missed in the search strategy. Authors of eligible studies were contacted to identify additional studies.

The search strategy followed the PICO (Population, Intervention, Comparison, Outcome) concept and focused on the two concepts “7-keto-DHEA” and “weight loss”. The search terms included abbreviations, synonyms, derivatives, and product names of 7-keto-DHEA. There exists no thesaurus term for 7-keto-DHEA yet. “DHEA” was added to the search concept to extend the sensitivity of the search results. The final detailed search strategy for the Medline Ovid database is shown in Table 1. This strategy was modified appropriately for the other databases and are presented in Online Resource 2.

**Table 1:** Search strategy for the Medline Ovid database (1946–27th October 2020)

#	Search terms	Results
1	Exp overweight/	222,620
2	(Overweight* or obese* or adipos*).tw	254,577
3	Body Weight/or body weight changes/ or weight loss/	223,687
4	((body adj3 weight*) or (weight adj3 change*) or weight-change* or (weight adj3 loss*) or weight-loss* or (weight adj3 reduct*) or weight-reduct* or antiobes* or anti-obes*).tw	320,451
5	Obesity management/ or weight reduction programs/	2383
6	Body constitution/ or exp body fat distribution/	25,461
7	(Body-fat-distribution or (body fat adj3 distribution) or body-fat-pattern* or (body fat adj3 pattern*) or fat load* or body lipid* or body-constitution or (body adj3 constitution) or body-composition or (body adj3 composition)).tw	42,789
8	Body mass index/or waist-hip ratio/or waist circumference/	132,990
9	(“Body mass index” or “body-mass index” or body-mass-index or bmi or “quetelet index” or “waist-hip-ratio” or “waist-hip ratio” or waist–hip-ratio or “waist-to-hip-ratio” or waist size* or (waist* adj3 size*) or hip circumference* or hip-circumference or “waist to hip ratio” or (waist* adj3 cirumference*)).tw	253,061
10	Energy metabolism/or basal metabolism/	88,472
11	(Metabolic rate* or energy metabolism* or basal metabolism* or energy expenditure*).tw	74,879
12	Anti-obesity agents/	5055
13	or/1–12	998,977
14	Exp Dehydroepiandrosterone/ae, aa, me, ph, pd, tu	6174
15	(“7 keto” or 7-Keto or 7-Keto-DHEA or 3-acetyl-7-oxo-dehydroepiandrosterone or “3 acetyl 7 oxo dehydroepiandrosterone” or 3-acetyl-7-oxo-DHEA or “3 acetyl 7 oxo DHEA” or 7-oxo-DHEA or “7 oxo DHEA” or 7-oxodehydroepiandrosterone or “7 oxodehydroepiandrosterone” or 7-ketodehydroepiandrosterone or “7 ketodehydroepiandrosterone” or 7-oxoprasteron or “7 oxoprasteron” or 7-ketoprasteron or “7 ketoprasteron” or 3-acetyl-7-oxo-DHEA or prasterone or “3β-Hydroxyandrost-5-ene-7,17-dione” or “3beta-Hydroxyandrost-5-ene-7,17-dione” or “5-Androsten-3β-ol-7,17-dione” or “5-Androsten-3beta-ol-7,17-dione” or “3-acetoxyandrost-5-ene-7,17-dione” or 7?-hydroxy-dehydroepiandrosterone or 7?-OH-DHEA or 7?-hydroxy-dehydroepiandrosterone or 7alpha-OH-DHEA or 7alpha-hydroxy-dehydroepiandrosterone or 7-oxo-DHEA-3?-sulfate or “7 keto naturalean” or 7-keto naturalean or “Lean system 7” or HUM5007).rn,sy,nm,tw	603
16	14 or 15	6637
17	13 and 16	752
18	Exp animals/ not humans.sh	4,749,702
19	17 not 18	432

### Study selection

All identified citations were imported into EndNote and duplicates were removed. Two authors (N.J., S.M.) independently screened the title, abstract and key words of the search results based on the inclusion criteria outlined in Table 2. The full text of eligible studies was further examined in detail. Conflicts at all levels of screening were resolved through discussion.

**Table 2:** Inclusion criteria for study selection

Type of study	All clinical trials of 7-keto-DHEA for weight loss will be included in the review independent of study type. No restrictions are applied
Type of participant	All patients with overweight or obesity will be included. There will be no restrictions on gender, education, or any other formal criteria
Type of intervention	Patients who have been treated with 7-keto-DHEA or any other dietary supplements containing 7-keto-DHEA will be included in the experimental group. Neither the form of administration nor the duration and frequency of treatment is limited
Type of control	The control group will include patients treated with a placebo drug
Type of outcome	The primary outcomes will be changes in weight loss parameters such as body weight, body fat, and body mass index. Secondary outcomes are basal and resting metabolic rate

### Risk of bias assessment

The methodological quality of the selected studies was independently evaluated by two reviewers (N.J., P.S.) using the Jadad scale [17]. It is composed of five questions shown in Table 3 and the quality is considered as “high” when more than three items are satisfied [18]. Additionally, it was assessed whether an intention-to-treat analysis was conducted or not.

**Table 3:** Jadad scale for quality assessment

Items	Scoring the items	Kalman et al. [21]	Zenk et al. [20]	Zenk et al. [3]	Zenk et al. [5]
1) Was the study described as randomized?	Yes (1 point)/no (0 point)	Yes	Yes	Yes	Yes
2) Was the study described as double blind?	Yes (1 point)/no (0 point)	Yes	Yes	Yes	Yes
3) Was there a description of withdrawals and dropouts?	Yes (1 point)/no (0 point)	Yes	Yes	Yes	Yes
4) Was the randomization appropriate?	Yes (1 point)/no (-1 point)	Yot reported	Yes	Yes	Yes
5) Was the double-blinding appropriate?	Yes (1 point)/ no (-1 point)	Yes	Yes	Yes	Yes
Total score		4	5	5	5

### Data extraction

From each eligible study, one author (N.J.) extracted information and the relevant data were entered into Table 4, including the first author, publication year, country of publication, clinical trial design, participants’ mean age, baseline BMI and body weight, study size, duration of study, outcome measures and the dosage of 7-keto-DHEA treatment. The table was then checked by another investigator (P.S.) and any disagreement was obtained through negotiation.

**Table 4:** Characteristics of eligible studies

Source	Country	Study design	Participants’ mean age (year)	BMI (km/m^2^)	Body Weight (kg)	Study size 7-keto-DHEA/placebo	Duration (weeks)	Outcome variables	7-Keto-DHEA dosage (mg/d)
Kalman et al. [21]	USA	RCT	44.5	31.9	91.6	13/10	8	Body weight, body fat percentage	200 (2 × 100)
Zenk et al. [20]	USA	RCT	48.7	33.1	96.5	17/16	8	Body weight, BMI, body fat percentage, BMR	200 (2 × 100 mg)
Zenk et al. [3]	USA	RCT	39.2	32.9	97.5	19/16	8	Body weight, BMI, body fat percentage, RMR	102 (2 × 51)
Zenk et al. [5]	USA	RCT	38.5	32.2	93.7	40/40	5^a^	Body weight, BMI, body fat percentage, RMR	200 (2 × 2 × 50)

^a^Each of the three 7 day treatment period (7-keto-DHEA, HUM5007, placebo) was separated by a 7 day washout period

## Results

### Study selection

**Fig. 2:**
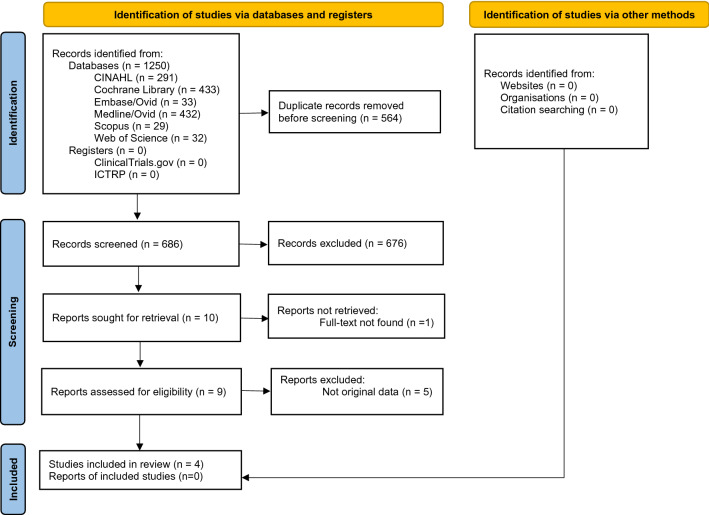
PRISMA 2020 flow diagram [19]

1250 records were retrieved from the various information sources. After removal of duplicates, 686 items were left to be screened from which ten papers were chosen for full-text examination. The full text of one paper could not be found. Another five papers were excluded because they were not clinical trials, leaving a final total of four studies [3, 5, 20, 21]. No additional records were extracted through hand-searching of reference lists or through contacting the authors of the remaining articles. The detailed steps of the study selection process are illustrated as a flow diagram in Fig. 2.

### Risk of bias assessment

The results of the quality assessment are shown in Table 3. Each study met the threshold for a low risk of bias after the Jadad scale. All four studies were randomized, double-blinded, placebo-controlled trials. Three studies reported a randomization table pre-coded by an independent statistician [3, 5, 20], whereas the method was not explicitly mentioned in one study [21]. The double blinding was ensured by indistinguishable capsules. A description of dropouts was given; however, each study had a dropout rate of above 5% and therefore might be attributed to attrition bias [22]. Furthermore, only one conducted an intention-to-treat analysis [3], while the rest used a complete case analysis [5, 20, 21].

### Study characteristics

The general characteristics of the four studies are depicted in Table 4. Collectively, they enrolled 131 participants with ages ranging from 20 to 70 years. Both sexes were included, with women in the majority. The treated and placebo groups did not differ significantly at baseline. The studied population comprised subjects with a BMI > 27 kg/m^2^ [3, 20, 21], except for a study [5] that enrolled subjects with BMI $\ge$ 25 kg/m^2^. Exclusion criteria for the population included a history of renal, hepatic, or cardiovascular diseases, cancer, diabetes mellitus, thyroid diseases, eating disorders, or the presence of any illness that might cause weight loss. Individuals were also excluded if they were pregnant, lactating, or using any medications for weight loss. The trials were published from 2000 to 2007 and were all conducted in the USA. The duration ranged from 1week [5] to 8 weeks [3, 20, 21]. Subjects were either given 7-keto-DHEA [5, 21] and/or a formula^1^ that, among others, contained 7-keto-DHEA [3, 5, 20]. Twice a day, 7-keto-DHEA was administered orally as its acetyl derivative 3-acetyl-7-keto-DHEA with the prescribed dose varying between 102 and 200 mg. Three studies required their subjects to follow a diet and an exercise program [3, 20, 21], whereas the fourth study prescribed only a calorie-restricted diet [5]. The placebo group either received maltodextrin [20, 21] or rice powder [3, 5]. No significant differences were found between both groups regarding dietary, exercise or drug compliance. The outcome variables were measured as mean differences at baseline, week 4 and 8 [3, 20, 21], except for one study, which evaluated at baseline and at the beginning and end of each treatment [5].

### Effect on body weight, body mass index, and body fat percentage

The effect of 7-keto-DHEA on body weight was measured with either a balanced medical scale [21] or a digital scale [3, 5, 20]. Two out of four studies revealed a statistically significant reduction in body weight when compared to placebo: Kalman et al. [21] mentioned a 4.4% weight loss after 8 weeks, meaning a weight loss of 2.88 kg (*P* = 0.01), and Zenk et al. [20] stated a weight reduction of 2.15 kg respectively—2.2% (*P* = 0.038). Zenk et al. [3, 5, 20] evaluated the impact of 7-keto-DHEA on BMI; however, only Zenk et al. [20] found a significant decrease of 0.71 (*P* = 0.036). The effect on body fat percentage was determined using a skinfold caliper [21], a bioelectrical impedance analysis [5, 20], or a dual-energy X-ray absorptiometry [3]. Only one study showed a significant reduction of 1.8% (*P* = 0.02) [21]. The results regarding body weight, BMI, and body fat percentage are depicted in Table 5, 6, and 7. Kalman et al. [21] and Zenk et al. [5] both provided outcome measures without standard deviations.

**Table 5:** Mean changes from baseline in body weight

Study	Intervention	Mean change $\pm$ STD treatment group	Mean change $\pm$ STD control group	*P* value
Kalman et al. [21]	7-Keto-DHEA	− 2.88 kg	− 0.97 kg	0.01
Zenk et al. [20]	7-Keto Naturalean	− 2.15 $\pm$ 2.38 kg	− 0.72 $\pm$ 2.12 kg	0.038
Zenk et al. [3]	Lean System 7	− 2.26 $\pm$ 2.44 kg	− 2.34 $\pm$ 3.12 kg	0.93
Zenk et al. [5]	7-Keto-DHEA	− 0.38 kg	− 0.55 kg	≥ 0.05
	HUM5007	− 0.56 kg	− 0.55 kg	≥ 0.05

**Table 6:** Mean changes from baseline in BMI

Study	Intervention	Mean change $\pm$ STD treatment group	Mean change $\pm$ STD control group	*P* value
Kalman et al. 2000 [21]	7-Keto-DHEA	Not measured	Not measured	Not measured
Zenk et al. 2002 [20]	7-Keto Naturalean	− 0.71 $\pm$ 0.79 kg/m^2^	− 0.01 $\pm$ 1.05 kg/m^2^	0.036
Zenk et al. 2005 [3]	Lean System 7	− 0.76 $\pm$ 0.86 kg/m^2^	− 0.78 $\pm$ 1.07 kg/ ^2^	0.93
Zenk et al. 2007 [5]	7-Keto-DHEA	− 0.11 kg/m^2^	− 0.19 kg/m^2^	≥0.05
	HUM5007	− 0.22 kg/m^2^	− 0.19 kg/m^2^	≥0.05

**Table 7:** Mean changes from baseline in body fat percentage

Study	Intervention	Mean change $\pm$ STD treatment group	Mean change $\pm$ STD control group	*p* value
Kalman et al. 2000 [21]	7-Keto-DHEA	− 1.80%	− 0.57%	0.02
Zenk et al. 2002 [20]	7-Keto Naturalean	− 0.58 $\pm$ 1.49%	− 0.01 $\pm$ 1.54%	0.41
Zenk et al. 2005 [3]	Lean System 7	− 0.72 $\pm$ 1.40 kg	− 0.38 $\pm$ 1.82 kg	0.47
Zenk et al. 2007 [5]	7-Keto-DHEA	− 0.50%	0.04%	≥ 0.05
	HUM5007	+ 0.15%	0.04%	≥ 0.05

### Effect on basal metabolic rate (BMR) and resting metabolic rate (RMR)

An indirect calorimetry was used by Zenk et al. [3, 5, 20] to measure the effect on either BMR or RMR, which are shown in Tables 8 and 9. Zenk et al. [5] showed a significant increase of 1.43% with 7-keto-DHEA and 3.4% with HUM5007 compared to placebo (*P* = 0.001); however, the difference in increase was not significant between both these treatment groups. Zenk et al. [3] noted a 7.2% increase. No significant changes in BMR were reported by Zenk et al. [20].

**Table 8:** Mean changes from baseline in RMR

Study	Intervention	Mean change $\pm$ STD treatment group	Mean change $\pm$ STD control group	*p* value
Kalman et al. 2000 [21]	7-Keto-DHEA	Not measured	Not measured	Not measured
Zenk et al. 2002 [20]	7-Keto Naturalean	Not measured	Not measured	Not measured
Zenk et al. 2005 [3]	Lean System 7	7.2 $\pm$ 1.6%	− 0.7 $\pm$ 0.84%	0.03
Zenk et al. 2007 [5]	7-Keto-DHEA	+ 1.43%	− 3.93%	0.001
	HUM5007	+ 3.36%	− 3.93%	0.001

**Table 9:** Mean changes from baseline in BMR

Study	Intervention	Mean change $\pm$ STD treatment group	Mean change $\pm$ STD control group	*p*-value
Kalman et al. 2000 [21]	7-Keto-DHEA	Not measured	Not measured	Not measured
Zenk et al. 2002 [20]	7-Keto Naturalean	+ 14.78 $\pm$ 662.35 kJ/d	− 7.82 $\pm$ 775.96 kJ/d	0.86
Zenk et al. 2005 [3]	Lean System 7	Not measured	Not measured	Not measured
Zenk et al. 2007 [5]	7-Keto-DHEA	Not measured	Not measured	Not measured
	HUM5007	Not measured	Not measured	Not measured

### Safety and tolerability

7-Keto-DHEA and the formulas were generally well tolerated. No serious adverse side effects were reported, and none resulted in discontinuation from the trial. The relatively few side effects that are possibly related to the treatment with 7-keto-DHEA include nausea and vertigo. The formulas reported the following adverse effects: nausea, urticaria, metallic taste, heart burn, flatulence, and palpitation. The studies found no significant changes in vital signs (systolic and diastolic blood pressure, pulse rate, temperature), liver and renal function, hematological profile (including blood sugar, testosterone, and estradiol levels), or in mental or emotional functioning. Kalman et al. [21] reported an increase in triiodothyronine (T3) within the normal range; however, the two newer studies [3, 20] observed no such increase in T3 levels.

## Discussion

While the weight loss ability of 7-keto-DHEA is highly promoted commercially, there is limited academic literature about 7-keto-DHEA in terms of weight loss. In the present literature review, 4 out of 686 papers retrieved from the literature search were eligible for reviewing 7-keto-DHEA and its effect on weight loss. However, three research papers were led by the same author. Moreover, only one of them focused exclusively on 7-keto-DHEA, while the others solely examined formulas containing 7-keto-DHEA. The four studies suggest a mixed result regarding weight loss. Zenk et al. [5] argued that the chosen treatment period is too short to determine the effect of 7-keto-DHEA on the outcome variables. This was supported by the findings of Kalman et al. [21], where no changes in body fat percentage were found at week 4, but significant differences were measured at week 8.

The four reviewed studies stated a low side-effect profile which is in accordance with the data presented by Davidson et al. [23] who investigated the safety of orally given 3-acetyl-7-oxo-DHEA in humans. While humans tolerated 7-keto-DHEA well at doses up to 200 mg per kg body weight [23], it was not toxic to rats at doses as high as 2000 mg per kg body weight [24] and to monkeys up to 500 mg/kg of body weight. No DNA alterations were found [25] and there were no known drug interactions [23]. Many therapeutically useful steroids are administered as acetyl-esters to protect against oxidation during synthesis [26] and are rapidly hydrolyzed in vivo to free steroids due to the widespread activity of human tissue esterase [13]. The administered 3-acetyl-7-oxo-DHEA is rapidly absorbed and exists predominantly as its sulfate conjugate in circulating blood. It is rapidly eliminated with a half-life of 2.17 h and thus does not accumulate in the body over time. Vital signs and blood hormone concentrations remained in the normal range [23]. This is contradicted by the findings of Sulcovà et al. [27] who reported immediate and delayed changes in blood hormone parameters after administering 7-keto-DHEA transdermally for eight consecutive days. The decrease in estradiol and testosterone may be specific for percutaneous application or is possibly attributed to the unconjugated form of 7-keto-DHEA [27]. In comparison to its parent hormone, 7-keto-DHEA is not further converted to androgen [7] and is not aromatized because the carbonyl group on the position 7 prevents aromatase from binding and converting it to estrogen [28]. Therefore, the side effects of DHEA seen with elevated testosterones or estrogens are said to be of no concern for 7-keto-DHEA [23]. Both, Davidson et al. [23] and Sulcovà et al. [27], examined the effects of 7-keto-DHEA on healthy male volunteers, thus leaving the effects on women in uncertainty.

The inclusion criteria for the population in the reviewed studies show a similar weakness. The studies excluded participants with previous illnesses, even though obesity is associated with various health conditions [2]. Moreover, pregnant and lactating women were excluded from the studies, although postpartum weight retainment increases the likeliness of obesity [29]. This highlights the need for more randomized studies to evaluate the full potential of 7-keto-DHEA. In 2017, the U.S. Food and Drug Administration proposed to not add 7-keto-DHEA to the list of bulk drug substances under 503A because of lacking clinical evidence regarding its safety and efficacy, particularly with prolonged use [30]. People who choose to ingest 7-keto-DHEA should be concerned with unsubstantiated claims, questionable quality control, and safety of long-term use. Further research should specifically address the limitations noted in all these studies, which include the small sample, the short study and follow-up duration, and sex and age differences in the study population.

## Conclusion

Currently available clinical studies are giving mixed answers regarding 7-keto-DHEA and weight loss. Further studies are encouraged before any therapeutical use can be recommended.

## Supplementary Information

Below is the link to the electronic supplementary material.

Supplementary file1 Database Search Strategies. (PDF 192 KB)

Supplementary file2 PRISMA checklist [16] (PDF 72 KB)

## Footnotes

1 *HUM5007* contains 7-keto-DHEA, calcium citrate, reen tea extract, ascorbic acid, chromium nicotinate, and cholecalciferol. *7-keto Naturalean* contains 7-keto-DHEA, L-tyrosine, asparagus root extract, choline bitartrate, inositol, copper gluconate, manganese, and potassium iodide. *Lean System 7* contains 7-keto-DHEA, yerba mate, guarana, citrus aurantium, coleus forskohlii, dandelion leaf, and piper nigrum.
