# Supplementary material for: A systematic review of the impact of 7-keto-DHEA on body weight
Source: Arch Gynecol Obstet. 2022 Dec 25;308(3):777–85. doi: 10.1007/s00404-022-06884-8 (PMC10348924; doi:10.1007/s00404-022-06884-8)
Supplement: Supplementary file 1 — Supplementary file1 Database Search Strategies. (PDF 192 KB) [file 404_2022_6884_MOESM1_ESM.pdf]

Systematic Review – the impact of 7-keto-DHEA on body weight

Archives of Gynecology and obstetrics
Nishanthini Jeyaprakash, Sara Maeder, Heidrun Janka, Petra Stute

Professor Dr. med. Petra Stute, M.D.

Department of Gynecologic Endocrinology and Reproductive Medicine

University Clinic of Obstetrics and Gynecology, Inselspital Bern

Friedbühlstrasse 19

3010 Bern, Switzerland

Tel: (0)31-632-1303

E-mail: petra.stute@insel.ch

ORCiD: 0000-0002-5591-1552

Database Search Strategies for Appendix

Date searched: October 28, 2020

****

MEDLINE via Ovid (1946-Present)

Database: Ovid MEDLINE(R) and Epub Ahead of Print, In-Process & Other Non-Indexed Citations, Daily and Versions(R) <1946 to October 27, 2020>
Search Strategy:
----
1 exp Overweight/ (222620)
2 (overweight* or obese* or adipos*).tw. (254577)
3 Body Weight/ or body weight changes/ or weight loss/ (223687)
4 ((body adj3 weight*) or (weight adj3 change*) or weight-change* or (weight adj3 loss*) or weight-loss* or (weight adj3 reduct*) or weight-reduct* or antiobes* or anti-obes*).tw. (320451)
5 Obesity Management/ or Weight Reduction Programs/ (2383)
6 Body Constitution/ or exp Body Fat Distribution/ (25461)
7 (body-fat-distribution or (body fat adj3 distribution) or body-fat-pattern* or (body fat adj3 pattern*) or fat load* or body lipid* or body-constitution or (body adj3 constitution) or body-composition or (body adj3 composition)).tw. (42789)

8 body mass index/ or Waist-Hip Ratio/ or Waist Circumference/ (132990)
9 ("body mass index" or "body-mass index" or body-mass-index or bmi or "quetelet index" or "waist-hip-ratio" or "waist-hip ratio" or waist-hip-ratio or "waist-to-hip-ratio" or waist size* or (waist* adj3 size*) or hip circumference* or hip-circumference or "waist to hip ratio" or (waist* adj3 cirumference*)).tw. (253061)
10 energy metabolism/ or basal metabolism/ (88472)
11 (metabolic rate* or energy metabolism* or basal metabolism* or energy expenditure*).tw. (74879)
12 Anti-Obesity Agents/ (5055)
13 or/1-12 (998977)
14 exp Dehydroepiandrosterone/ae, aa, me, ph, pd, tu (6174)
15 ("7 keto" or 7-Keto or 7-Keto-DHEA or 3-acetyl-7-oxo-dehydroepiandrosterone or "3 acetyl 7 oxo dehydroepiandrosterone" or 3-acetyl-7-oxo-DHEA or "3 acetyl 7 oxo DHEA" or 7-oxo-DHEA or "7 oxo DHEA" or 7-oxodehydroepiandrosterone or "7 oxodehydroepiandrosterone" or 7-ketodehydroepiandrosterone or "7 ketodehydroepiandrosterone" or 7-oxoprasteron or "7 oxoprasteron" or 7-ketoprasteron or "7 ketoprasteron" or 3-acetyl-7-oxo-DHEA or prasterone or "3β-Hydroxyandrost-5-ene-7,17-dione" or "3beta-Hydroxyandrost-5-ene-7,17-dione" or "5-Androsten-3β-ol-7,17-dione" or "5-Androsten-3beta-ol-7,17-dione" or "3-acetoxyandrost-5-ene-7,17-dione" or 7?-hydroxy-dehydroepiandrosterone or 7?-OH-DHEA or 7?-hydroxy-dehydroepiandrosterone or 7alpha-OH-DHEA or 7alpha-hydroxy-dehydroepiandrosterone or 7-oxo-DHEA-3?-sulfate or "7 keto naturalean" or 7-keto naturalean or "Lean system 7" or HUM5007).rn,sy,nm,tw. (603)
16 14 or 15 (6637)
17 13 and 16 (752)
18 exp animals/ not humans.sh. (4749702)
19 17 not 18 (432)

****

Embase via Ovid (1974-Present)

Database: Embase <1974 to 2020 October 27>
Search Strategy:
----
1 exp obesity/ (529715)
2 (overweight* or obese* or adipos*).ti,ab,kw. (374453)
3 body weight/ or exp body weight change/ or body weight loss/ or body weight control/ or body weight disorders/ (359204)
4 ((body adj3 weight*) or (weight adj3 change*) or weight-change* or (weight adj3 loss*) or weight-loss* or (weight adj3 reduct*) or weight-reduct* or antiobes* or anti-obes*).ti,ab,kw. (458296)
5 exp body weight management/ (4916)
6 exp body composition/ or body constitution/ (109398)
7 (body-fat-distribution or (body fat adj3 distribution) or body-fat-pattern* or (body fat adj3 pattern*) or fat load* or body lipid* or body-constitution or (body adj3 constitution) or body-composition or (body adj3 composition)).ti,ab,kw. (61153)
8 body mass/ or Waist-Hip Ratio/ or Waist Circumference/ (476891)

9 ("body mass index" or "body-mass index" or body-mass-index or bmi or "quetelet index" or "waist-hip-ratio" or "waist-hip ratio" or waist-hip-ratio or "waist-to-hip-ratio" or waist size* or (waist* adj3 size*) or hip circumference* or hip-circumference or "waist to hip ratio" or (waist* adj3 cirumference*)).ti,ab,kw. (463316)
10 exp energy metabolism/ or exp basal metabolic rate/ (208306)
11 (metabolic rate* or energy metabolism* or basal metabolism* or energy expenditure*).ti,ab,kw. (94565)
12 exp antiobesity agent/ (4966)
13 or/1-12 (1684872)
14 prasterone/ (15119)
15 ("7 keto" or 7-Keto or 7-Keto-DHEA or 3-acetyl-7-oxo-dehydroepiandrosterone or "3 acetyl 7 oxo dehydroepiandrosterone" or 3-acetyl-7-oxo-DHEA or "3 acetyl 7 oxo DHEA" or 7-oxo-DHEA or "7 oxo DHEA" or 7-oxodehydroepiandrosterone or "7 oxodehydroepiandrosterone" or 7-ketodehydroepiandrosterone or "7 ketodehydroepiandrosterone" or 7-oxoprasteron or "7 oxoprasteron" or 7-ketoprasteron or "7 ketoprasteron" or 3-acetyl-7-oxo-DHEA or prasterone or "3β-Hydroxyandrost-5-ene-7,17-dione" or "3beta-Hydroxyandrost-5-ene-7,17-dione" or "5-Androsten-3β-ol-7,17-dione" or "5-Androsten-3beta-ol-7,17-dione" or "3-acetoxyandrost-5-ene-7,17-dione" or 7?-hydroxy-dehydroepiandrosterone or 7alpha-hydroxy-dehydroepiandrosterone or 7?-OH-DHEA or 7alpha-OH-DHEA or 7-oxo-DHEA-3?-sulfate or "7 keto naturalean" or 7-keto naturalean or "Lean system 7" or HUM5007).tn,ti,ab,kw. (626)
16 14 or 15 (15479)
17 13 and 15 (56)
18 limit 17 to human (33)

****

Cochrane Library (1996-Present)

#1	MeSH descriptor: [Overweight] explode all trees	(16289)
#2	(overweight* or obese* or adipos*):ti,ab,kw	(34805)
#3	MeSH descriptor: [Body Weight] this term only	(8356)
#4	MeSH descriptor: [Body Weight Changes] this term only	(87)
#5	MeSH descriptor: [Weight Loss] explode all trees	(6327)
#6	(body weight* or body-weight* or weight loss* or weight-loss* or weight change* or weight-change* or weight reduct* or weight-reduct* or antiobes* or anti-obes*):ti,ab,kw	(84676)
#7	MeSH descriptor: [Obesity Management] this term only	(11)
#8	MeSH descriptor: [Weight Reduction Programs] this term only	(730)
#9	MeSH descriptor: [Body Constitution] this term only	(264)
#10	MeSH descriptor: [Body Fat Distribution] explode all trees	(905)
#11	(body-fat-distribution* or body fat distribution* or body-fat-pattern* or body fat pattern* fat load* or body lipid* or body-constitution* or body constitution* or body-composition or body composition*):ti,ab,kw	(25645)
#12	MeSH descriptor: [Body Mass Index] explode all trees	(10061)
#13	MeSH descriptor: [Waist-Hip Ratio] explode all trees	(257)
#14	MeSH descriptor: [Waist Circumference] explode all trees	(1025)

#15 (body mass index* or body-mass index* or body-mass-index or bmi or quetelet index* or waist-hip-ratio or waist-hip ratio* or waist-hip-ratio or waist-to-hip-ratio or hip circumference* or waist size*):ti,ab,kw (70457)
#16 MeSH descriptor: [Energy Metabolism] this term only (3232)
#17 MeSH descriptor: [Basal Metabolism] this term only (454)
#18 (metabolic rate* or energy metabolism* or basal metabolism* or energy expenditure*):ti,ab,kw (25423)
#19 MeSH descriptor: [Anti-Obesity Agents] this term only (453)
#20 #1 or #2 or #3 or #4 or #5 or #6 or #7 or #8 or #9 or #10 or #11 or #12 or #13 or #14 or #15 or #16 or #17 or #18 or #19 (155959)
#21 MeSH descriptor: [Dehydroepiandrosterone] explode all trees (677)
#22 ((7 NEAR/2 Keto DHEA) or (7 NEAR/2 keto) or (3 NEXT acetyl 7 NEAR/2 oxo dehydroepiandrosterone) or (3 NEAR/2 acetyl 7 oxo DHEA) or (7 NEAR/2 oxo DHEA) or (7 NEAR/2 oxodehydroepiandrosterone) or (7 NEAR/2 ketodehydroepiandrosterone) or (7 NEAR/2 oxoprasteron) or (7 NEAR/2 ketoprasteron) or prasterone or (7 NEAR/2 OH DHEA) or (7 NEAR/2 hydroxy-dehydroepiandrosterone) or (7 NEAR/2 oxo DHEA 3 sulfate) or (7 NEAR/2 keto naturalean) or (Lean system 7) or HUM5007):ti,ab,kw (500)
#23 #21 or #22 (1077)
#24 #20 and #23 (433)

****

CINAHL (1937 - Present)

#17	291	S15 AND S16
#16	1,469	S13 OR S14
#15	310,198	S1 OR S2 OR S3 OR S4 OR S5 OR S6 OR S7 OR S8 OR S9 OR S10 OR S11 OR S12
#14	60	TI ("7 keto" or 7-Keto or 7-Keto- DHEA or 3-acetyl-7-oxo-dehydroepiandrosterone or "3 acetyl 7 oxo dehydroepiandrosterone" or 3-acetyl-7-oxo-DHEA or "3 acetyl 7 oxo DHEA" or 7-oxo-DHEA or "7 oxo DHEA" or 7- oxodehydroepiandrosterone or "7 oxodehydroepiandrosterone" or 7-ketodehydroepiandrosterone or "7 ketodehydroepiandrosterone" or 7-oxoprasteron or "7 oxoprasteron" or 7- ketoprasteron or "7 ketoprasteron" or 3-acetyl-7-oxo-DHEA or prasterone or "3β-Hydroxyandrost-5-ene-7,17- dione" or "3beta-Hydroxyandrost-5-ene-7,17-dione" or "5-Androsten-3β-ol-7,17-dione" or "5-Androsten-3beta-ol-7,17-dione" or "3-acetoxyandrost-5- ene-7,17-dione" or 7?-hydroxy- dehydroepiandrosterone or 7alpha- hydroxy-

	dehydroepiandrosterone or 7?-OH-DHEA or 7alpha-OH-DHEA or 7-oxo-DHEA-3?-sulfate or "7 keto naturalean" or 7-keto naturalean or "Lean system 7" or HUM5007) OR AB ("7 keto" or 7-Keto or 7-Keto- DHEA or 3-acetyl-7-oxo- dehydroepiandrosterone or "3 acetyl 7 oxo dehydroepiandrosterone" or 3-acetyl-7-oxo-DHEA or "3 acetyl 7 oxo DHEA" or 7-oxo-DHEA or "7 oxo DHEA" or 7-oxodehydroepiandrosterone or "7 oxodehydroepiandrosterone" or 7-ketodehydroepiandrosterone or "7 ketodehydroepiandrosterone" or 7-oxoprasteron or "7 oxoprasteron" or 7- ketoprasteron or "7 ketoprasteron" or 3-acetyl-7-oxo-DHEA or prasterone or "3β-Hydroxyandrost-5-ene-7,17- dione" or "3beta-Hydroxyandrost-5- ene-7,17-dione" or "5-Androsten-3β- ol-7,17-dione" or "5-Androsten-3beta- ol-7,17-dione" or "3-acetoxyandrost-5- ene-7,17-dione" or 7?-hydroxy- dehydroepiandrosterone or 7alpha-hydroxy-dehydroepiandrosterone or 7?-OH-DHEA or 7alpha-OH-DHEA or 7-oxo-DHEA-3?-sulfate or "7 keto naturalean" or 7-keto naturalean or "Lean system 7" or HUM5007)
#13 1,448	(MH "Dehydroepiandrosterone")
#12 1,800	(MH "Antiobesity Agents")
#11 10,538	TI ("metabolic rate" or "energy metabolism" or "basal metabolism" or "energy expenditure") OR AB ("metabolic rate" or "energy metabolism" or "basal metabolism" or "energy expenditure")
#10 16,502	(MH "Energy Metabolism") OR (MH "Basal Metabolism+")
#9 89,042	TI ("body mass index" or "body-mass index" or bmi or "quetelet index" or "waist-hip-ratio" or "waist-hip ratio" or "waist-to-hip-ratio" or "waist size" or "hip circumference" or hip- circumference or "waist to hip ratio") OR AB ("body mass index" or "body- mass index" or bmi or "quetelet index" or "waist-hip-ratio" or "waist-hip ratio" or "waist-to-hip-ratio" or waist size* or "hip circumference" or hip- circumference or "waist to hip ratio")
#8 86,387	(MH "Body Mass Index") OR (MH "Waist Circumference") OR (MH "Waist-Hip Ratio")
#7 27,423	TI (body-fat-distribution or "body fat distribution" or body-fat-pattern or "body fat pattern" or "fat load" or "body lipid" or body-constitution or "body constitution" or body- composition or "body composition" OR AB (body-fat-distribution or "body fat distribution" or body-fat-pattern" or "body fat pattern" or "fat load" or "body lipid" or body-constitution or "body constitution" or body-composition or "body composition")
#6 79,307	(MH "Body Constitution+")
#5 23,070	(MH "Body Composition+")
#4 112,709	TI ("body weight" OR weight-loss* OR "weight change" OR weight-change* or "weight loss" or weight-loss* or "weight reduction" or weight- reduction* or antiobes* or anti-obes* or overweight* or obese* or adipos*) OR AB ("body weight" OR weight- loss* OR "weight change" OR weight- change* or "weight

	loss" or weight- loss* or "weight reduction" or weight-reduction* or antiobes* or anti-obes* or overweight* or obese* or adipos*)
#3 52,478	(MH "Body Weight Changes") OR (MH "Weight Loss") OR (MH "Body Weight")
#2 70,781	TI (overweight* or obese* or adipos*) OR AB (overweight* or obese* or adipos*)
#1 83,164	(MH "Obesity")

****

Web of Science (1900-Present)

#7 **32**	#6 AND #5<br>Indexes=SCI-EXPANDED, SSCI, A&HCI, CPCI-S, CPCI-SSH, ESCI<br>Timespan=All years
#6 918,969	#4 OR #3 OR #2 OR #1<br>Indexes=SCI-EXPANDED, SSCI, A&HCI, CPCI-S, CPCI-SSH, ESCI<br>Timespan=All years
#5 573	TS=("7 keto" or 7-Keto or 7-Keto-DHEA or 3-acetyl-7-oxo-dehydroepiandrosterone or "3 acetyl 7 oxo dehydroepiandrosterone" or 3-acetyl-7-oxo-DHEA or "3 acetyl 7 oxo DHEA" or 7-oxo-DHEA or "7 oxo DHEA" or 7-oxodehydroepiandrosterone or "7 oxodehydroepiandrosterone" or 7-ketodehydroepiandrosterone or "7 ketodehydroepiandrosterone" or 7-oxoprasteron or "7 oxoprasteron" or 7-ketoprasteron or "7 ketoprasteron" or 3-acetyl-7-oxo-DHEA or prasterone or "3β-Hydroxyandrost-5-ene-7,17-dione" or "3beta-Hydroxyandrost-5-ene-7,17-dione" or "5-Androsten-3β-ol-7,17-dione" or "5-Androsten-3beta-ol-7,17-dione" or "3-acetoxyandrost-5-ene-7,17-dione" or 7?-hydroxy-dehydroepiandrosterone or 7alpha-hydroxy-dehydroepiandrosterone or 7?-OH-DHEA or 7alpha-OH-DHEA or 7-oxo-DHEA-3?-sulfate or "7 keto naturalean" or 7-keto naturalean or "Lean system 7" or HUM5007)<br>Indexes=SCI-EXPANDED, SSCI, A&HCI, CPCI-S, CPCI-SSH, ESCI<br>Timespan=All years
#4 105,385	TS= ("metabolic rate" or "energy metabolism" or "basal metabolism" or "energy expenditure")<br>Indexes=SCI-EXPANDED, SSCI, A&HCI, CPCI-S, CPCI-SSH, ESCI<br>Timespan=All years
#3 279,140	TS=("body mass index" or "body-mass-index" or bmi or "quetelet index" or

	"waist-hip-ratio" or "waist-hip ratio" or "waist-to-hip-ratio" or "waist size" or "hip circumference" or hip-circumference or "waist to hip ratio")<br>Indexes=SCI-EXPANDED, SSCI, A&HCI, CPCI-S, CPCI-SSH, ESCI<br>Timespan=All years
#2 74,862	TS=(body-fat-distribution or "body fat distribution" or body-fat-pattern* or "body fat pattern" or "fat load" or "body lipid" or body-constitution or "body constitution" or body-composition or "body composition")<br>Indexes=SCI-EXPANDED, SSCI, A&HCI, CPCI-S, CPCI-SSH, ESCI<br>Timespan=All years
#1 646,525	TS=(((body NEAR/1 weight*) or weight-loss* or (weight NEAR/1 change*) or weight-change* or (weight NEAR/1 loss*) or weight-loss* or (weight NEAR/1 reduct*) or weight-reduct* or antiobes* or anti-obes* or overweight* or obese* or adipos*))<br>Indexes=SCI-EXPANDED, SSCI, A&HCI, CPCI-S, CPCI-SSH, ESCI<br>Timespan=All years

****

Scopus (1788-Present)

((TITLE-ABS(overweight* or obese* or adipos*) OR TITLE-ABS((body W/3 weight) or body-weight* or weight-loss* or (weight W/3 loss*) or (weight W/3 change*) or weight-change* or (weight W/3 reduct*) or weight-reduct* or antiobes* or anti-obes*) OR TITLE-ABS(body-fat-distribution or (body W/3 fat W/3 distribution) or body-fat-pattern* or (body W/3 fat W/3 pattern*) or (fat W/1 load*) or (body W/1 lipid*) or body-constitution or (body W/3 constitution) or body-composition or (body W/3 composition)) OR TITLE-ABS("body-mass index" or body-mass-index or bmi or "quetelet index" or "waist-hip ratio" or waist-to-hip-ratio or (waist W/1 size*) or (waist* W/3 size*) or (hip W/1 circumference*) or hip-circumference) OR TITLE-ABS((metabolic W/1 rate*) or (energy W/1 metabolism*) or (basal W/1 metabolism*) or (energy W/1 expenditure*)))) AND ((TITLE-ABS("7 keto" or 7-Keto or 7-Keto-DHEA or 3-acetyl-7-oxo-dehydroepiandrosterone or "3 acetyl 7 oxo dehydroepiandrosterone" or 3-acetyl-7-oxo-DHEA or "3 acetyl 7 oxo DHEA" or 7-oxo-DHEA or "7 oxo DHEA" or 7-oxodehydroepiandrosterone) OR TITLE-ABS("7 oxodehydroepiandrosterone" or 7-ketodehydroepiandrosterone or "7 ketodehydroepiandrosterone" or 7-oxoprasteron or "7 oxoprasteron" or 7-ketoprasteron or "7 ketoprasteron" or 3-acetyl-7-oxo-DHEA or prasterone or "3β-Hydroxyandrost-5-ene-7,17-dione")))

29 Search results

The search in two international trial registers, Clinicaltrials.gov and ICTRP Database (WHO), did not yield any additional studies.

Results

Total records:	1250
Duplicates:	564
New total:	686
